# Fluorescence Resonance Energy Transfer Assay for High-Throughput Screening of ADAMTS1 Inhibitors 

**DOI:** 10.3390/molecules161210709

**Published:** 2011-12-20

**Authors:** Jianhao Peng, Lili Gong, Kun Si, Xiaoyu Bai, Guanhua Du

**Affiliations:** 1 Institute of Materia Medica, Chinese Academy of Medical Science & Peking Union Medical College, Beijing 100050, China; Email: pengjianhao@hotmail.com (J.P.); gonglili2010@hotmail.com (L.G.); bxy716@163.com (X.B.); 2 Department of Macromolecular Science and Engineering, Case Western Reserve University, Cleveland, OH 44106, USA; Email: kun.si1@case.edu (K.S.)

**Keywords:** ADAMTS1, inhibitor, high-throughput screening, fluorescence resonance energy transfer, drug discovery

## Abstract

A disintegrin and metalloprotease with thrombospondin type I motifs-1 (ADAMTS1) plays a crucial role in inflammatory joint diseases and its inhibitors are potential candidates for anti-arthritis drugs. For the purposes of drug discovery, we reported the development and validation of fluorescence resonance energy transfer (FRET) assay for high-throughput screening (HTS) of the ADAMTS1 inhibitors. A FRET substrate was designed for a quantitative assay of ADAMTS1 activity and enzyme kinetics studies. The assay was developed into a 50-µL, 384-well assay format for high throughput screening of ADAMTS1 inhibitors with an overall Z’ factor of 0.89. ADAMTS1 inhibitors were screened against a diverse library of 40,960 total compounds with the established HTS system. Four structurally related hits, naturally occurring compounds, kuwanon P, kuwanon X, albafuran C and mulberrofuran J, extracted from the Chinese herb *Morus alba* L., were identified for further investigation. The results suggest that this FRET assay is an excellent tool, not only for measurement of ADAMTS1 activity but also for discovery of novel ADAMTS1 inhibitors with HTS.

## 1. Introduction

A disintegrin and metalloprotease with thrombospondin type I motifs-1 (ADAMTS1) is a secretory extracellular matrix (ECM)-anchored metalloproteinase [1]. ADAMTS-1 was originally cloned as an inflammation-associated gene and has subsequently been shown to be involved in extracellular matrix remodeling during ovulation, wound healing and angiogenesis. ADAMTS1 also plays an important role in cartilage ECM metabolism during the development of cartilage and progression of joint diseases [2,3,4]. It consists of a proprotein, a metalloproteinase and a disintegrin-like domain, a central thrombospondin (TSP) type I motif, a spacing region, and C-terminal TSP motifs [5,6]. The TSP type I motif and the spacing region are important for the tight interaction with ECM [7]. Maturation of ADAMTS1 requires two independent and sequential processing events that release two forms of ADAMTS1, p87-ADAMTS1 (87 kDa) and p65-ADAMTS1 (65 kDa), respectively [8]. Both p87- and p65-ADAMTS1 display catalytic activity toward matrix proteoglycans [9].

ADAMTS1 is able to cleave the large aggregating proteoglycan family, such as aggrecan, versican and brevican, which are the major constituents of articular cartilage ECM together with type II collagen [10,11]. The degradation products have been detected in the synovial fluids of patients suffering from various joint diseases. Normal cartilage ECM is in a state of dynamic equilibrium between synthesis and degradation [12]. In diseases such as osteoarthritis (OA) and rheumatoid arthritis (RA), ECM degradation exceeds its synthesis, resulting in a net decrease in the amount of cartilage matrix or even in a complete erosion of the cartilage at the joint surface [13]. ADAMTS1 and associated family members may be key enzymes in degradation of cartilage leading to inflammation and arthritis. Compared to ADAMTS4 (aggrecanase 1) and ADAMTS5 (aggrecanase 2), the aggrecanase activity of ADAMTS1 is lower. However, its activity can be enhanced by the binding of cofactor such as fibulin 1 [14]. While current inflammatory joint diseases treatments provide only symptomatic relief using nonsteroidal anti-inflammatory drugs, there is no therapy available to halt and/or reverse the progression of these debilitating diseases. Therefore, targeting ADAMTS1 may be a useful therapeutic strategy for the prevention of cartilage degradation in inflammatory joint diseases [15,16,17].

In the current study, we describe a method for cloning, expression, purification and initial characterization of ADAMTS1 enzyme. The fluorescence resonance energy transfer (FRET) peptide substrate was synthesized and the ADAMTS1 activity was detected with the increase of fluorescence due to the cleavage of its substrate. This assay can be used to provide a rapid method for measuring ADAMTS1 enzyme activity and discovering ADAMTS1 inhibitors by high-throughput screening (HTS). Our results showed that this HTS assay was simple, sensitive and cost-effective. We screened 40,960 samples and identified four naturally occurring compounds extracted from the Chinese herb *Morus alba *L., as potential ADAMTS1 inhibitors.

## 2. Results and Discussion

### 2.1. Expression of Recombinant ADAMTS1 in E. coli

**Figure 1:**
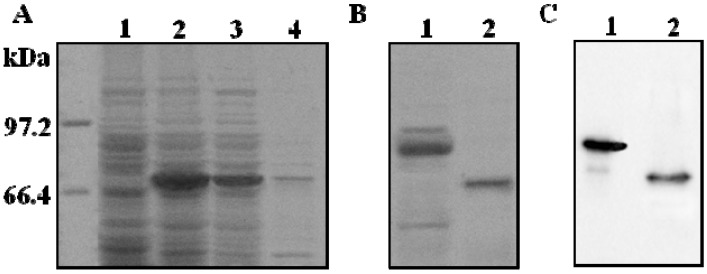
Expression analysis of recombinant ADAMTS1 in *E. coli* BL21 (DE3). (**A**) SDS-PAGE analysis of recombinant ADAMTS1 induced by IPTG. Lane 1: uninduced bacteria lysate; lane 2: IPTG wholly induced bacteria lysate; lane 3: supernatant of bacteria lysate; lane 4: precipitation of bacteria lysate; (**B**) SDS-PAGE analysis of purified fusion ADAMTS1 and ADAMTS1 on the Coomassie brilliant blue-stained gel; (**C**) Western blot analysis of purified fusion ADAMTS1 and ADAMTS1. Lane 1: purified fusion protein with NTA column; lane 2: the final purified protein after removal of thioredoxin using heparin-sepharose column.

*E. coli* BL21 (DE3) transformed by empty vector and recombinant vector pET32a-ADAMTS1 were cultured and induced with 1 mM IPTG. The expression of fusion protein is shown in Figure 1A. The target recombinant protein p65-ADAMTS1, with a molecular weight around 70 kDa, was only expressed by *E. coli*. The molecular weight of this recombinant protein is higher than the native one since it contains Trx and His tag. The quantified intensity of protein bands on SDS-PAGE gel showed that the recombinant ADAMTS1 accounts for about 43% of the total protein in the crude lysate. It was revealed that the recombinant ADAMTS1 was overexpressed in *E. coli* in a favorable way over non-target proteins. More than 70% of the recombinant protein was present in the *E. coli* BL21 supernatant after sonication lysed, suggesting that the ADAMTS1 was mainly soluble and located in cytoplasm, but not in the inclusion bodies. 

### 2.2. Purification and Proteolytic Cleavage of ADAMTS1 Fusion Protein

The supernatant was applied to a Ni-NTA affinity column to allow the binding between his·tagged ADAMTS1 recombinant protein and nickel beads. The fusion protein was eluted from the column with 300 mM imidazole [18] and the purity of the fusion protein reached 83%. ADAMTS1 fusion protein was incubated with enterokinase for 15 h at 25 °C. Fusion protein was found to be cleaved as indicated in Figure 1B. Previous research showed that the TSP motifs at the C terminus of ADAMTS1 were important for heparin binding and likely to be the sites which confer heparin affinity to ADAMTS1 [19]. The fractions containing ADAMTS1 proteins were applied to a heparin-sepharose column. Bound proteins were eluted with 20 mM PBS buffer containing 500 mM NaCl. The eluted fractions were analyzed by 10% SDS-PAGE gel. The purity of the final protein reached around 96%. The expressed ADAMTS1 was identified by Western blot analysis as shown in Figure 1C. The results showed that the ADAMTS1 was pure and sufficient for the high throughput screening.

### 2.3. Properties of Recombinant ADAMTS1 Using the FRET Peptide

**Figure 2:**
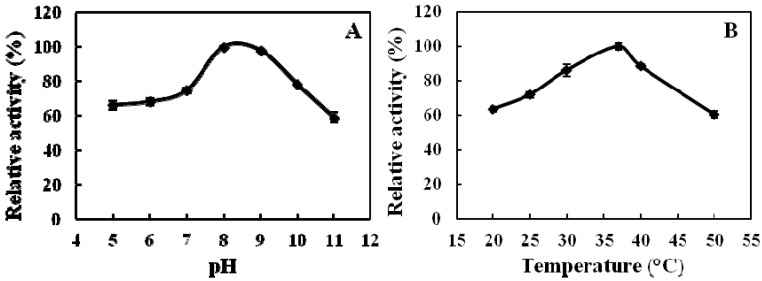
Effects of pH (**A**) and temperature (**B**) on the activity of ADAMTS1. The relative activity is expressed as the percentage of the maximum activity. (**A**) For determining the optimal pH of the ADAMTS1, the enzyme activity was measured under the enzyme assay conditions; (**B**) For determining the optimal temperature of the ADAMTS1, assays were carried out at various temperatures under the enzyme assay conditions. n = 3, Means ± SD.

We synthesized the intramolecularly quenched fluorescent substrate containing the *o*-aminobenzoic acid (Abz) group as the fluorescent donor, and 2,4-dinitrophenyl (Dnp) attached to the ε-amino group of lysine, as the fluorescent acceptor. Recombinant ADAMTS1 is able to hydrolyze the substrate, Abz-YPLPRNITEGEARGNVILTAK(Dnp)P-OH, at the E-A bond [11]. The effects of pH and temperature on the cleavage activity of the purified ADAMTS1 are shown in Figure 2. The enzyme activity reached its highest at pH 8.0, with an observation of significant stability in the range of pH 7.0–9.0. The enzyme activity decreased dramatically below pH 6.0 and above pH 9.0. The optimal temperature for the enzyme activity was 37 °C. Similarly, considerable stability was noticed in the range of 30–40 °C. Subsequently, all standard enzyme assays were carried out at pH 8.0 and 37 °C. 

**Figure 3:**
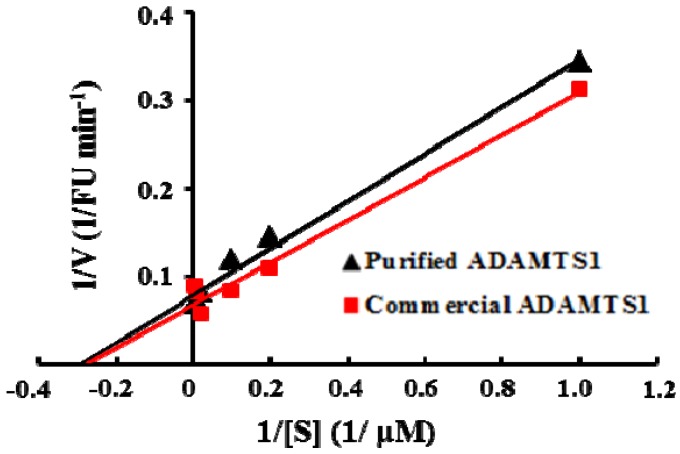
Lineweaver-Burk plots for purified *E. coli* and mammalian cell expressed ADAMTS1 at 0, 10, 25, 50, 100 and 150 µM concentrations of the substrate peptide.

According to the Michaelis-Menten theory of enzyme action, we measured the steady-state kinetic parameters *K*m and *V*max of the enzyme with increasing concentrations of the substrate under the optimal conditions. As shown in Figure 3, the *K*m and *V*max of *E. coli*-expressed ADAMTS1 for this FRET-based assay were calculated by Lineweaver-Burk plot as 3.38 μM and 12.64 FU min^−1^, respectively. Using the human recombinant ADAMTS1 expressed in the murine myeloma cell line, the *K*m and *V*max was 3.19 μM and 13.52 FU min^−1^. It indicates that ADAMTS1 expressed in *E. coli *has the similar kinetic activity compared with mammalian cell-expressed ADAMTS1. *E. coli *is the most frequently used prokaryotic expression system for production of heterologous proteins due to its efficiency, cost-effectiveness and potential for high-level production. Since HTS need large amounts of a highly purified enzyme, we successfully developed the soluble recombinant expression strategy to express functional ADAMTS1 in *E. coli*. To adapt the assay for screening in a 384-well plate format, a series of controls were performed to optimize the assay conditions. This assay was performed with substrate concentration at the *K*m value 3.38 μM which is the half maximal initial velocity. When the ADAMTS1 concentration was 10 nM, we observed the reaction linearity up to 30 min. DMSO is usually employed as universal solvent for new compounds in drug assay. In this study, compounds were dissolved in 1% DMSO and the interference of DMSO with the assay was negligible (data not shown). Therefore, 1% DMSO was applied in the routine screening without taking into account the solvent interference.

### 2.4. FRET-Based High-Throughput Drug Screening

**Figure 4:**
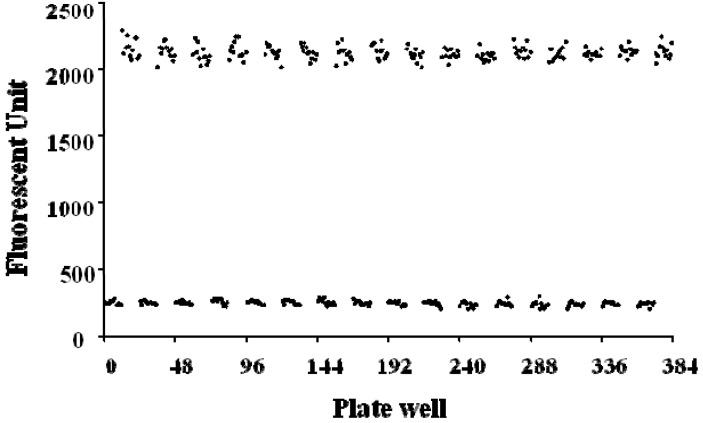
Z’ factor plot of high-throughput screening of ADAMTS1 inhibitors using the FRET assay in the black 384-well plates.

After establishing the initial enzymatic controls, we evaluated the statistical confidence of the analysis methods in the high-throughput drug screening application, based on the variation associated with individual measurements and the dynamic range of the system, the Z’ factor was calculated from uncleaved and maximal cleaved substrate and shown in Figure 4. 

The basal signals (signal in the absence of enzyme) for the 196 replicates were 244.3 ± 16.6. The maximal signals (signal after enzyme reaction) for the 196 replicates were 2,119.5 ± 52.5, which were quite high with a little variation. As a result, the detection window (difference between the maximal and basal readings) was good with the signal-to-noise ratio about 10. The Z’ factor was calculated to evaluate the quality of the overall assay. Here, the Z’ factor of the assay was 0.89, which indicated a stable and excellent system. Thus, this system is suitable for a high-throughput screening of ADAMTS1 inhibitors. 

**Figure 5:**
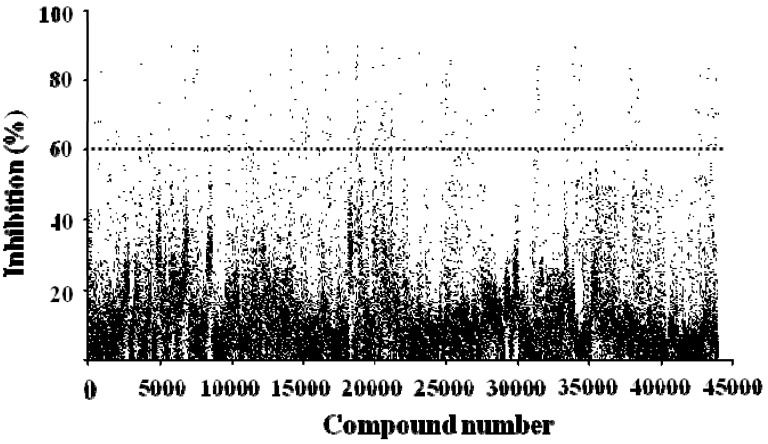
Summary of high-throughput screening of 40,960 compounds for inhibition of ADAMTS1 activity. Each dot represents one compound.

A diverse library of 40,960 compounds was examined for their inhibitory profile on ADAMTS1 activity in the primary screening process (Figure 5). Five µL of each compound at the concentration of 10 µg/mL was added to each of black 384-well plates. Compounds showing more than 60% inhibition (248) were identified and subjected to secondary screening under the same conditions to limit uncertainty. Four of them, J14713, J14714, J14715 and J14716, extracted from the Chinese herb *Morus alba* L., were validated as hits. The effective inhibitory concentrations of these four compounds were further investigated. 

### 2.5. Evaluation of Four Hit compounds

All assay detection methods will generate some artifacts due to compound interference, resulting in false-positive hits in the primary hit lists. These hit compounds with increasing concentrations were incubated with *E. coli* and mammalian expressed ADAMTS1 and substrate for 30 min to determine their 50% inhibition values (IC_50_), which was used to eliminate most of those detection artifacts and nonspecific hits. The structure and IC_50_ values of these four natural compounds are shown in Table 1. All four are polyphenolic compounds extracted from the root bark of cultivated mulberry tree (*Morus alba* L.), the Chinese herb “Sang-Bai-Pi”. Their structures were all identified in the literature and named as kuwanon P (J14713), kuwanon X (J14714), albafuran C (J14715) and mulberrofuran J (J14716) [20,21,22,23]. These compounds can be regarded biogenetically as Diels-Alder type adducts of chalcone derivatives and dehydroprenylphenols. All four compounds are optically active, with three chiral centers (3″*S*, 4″*S*, 5″*R*) on the methylcyclohexene ring. The triple chirality and multiple hydroxyl groups in the molecules probably account for the high inhibition ability towards ADAMTS1. Among them, kuwanon P and albafuran C showed significant lower IC_50_ values than kuwanon X and mulberrofuran J, which might be caused by the lack of steric hindrance at the 3″*S* chiral center in the former two compounds compared with the latter two compounds having an extra hydroxyl group nearby.

**Table 1:** Chemical structure and IC_50_ of hit compounds. (IC_50_ ± standard error of the mean of three independent experiments).

Compound	Structure	IC_50_ (μM) *	IC_50_ (μM) ^#^
J14713 Kuwanon P	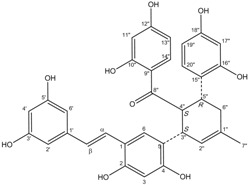	7.6 ± 0.6	5.5 ± 0.4
J14714 Kuwanon X	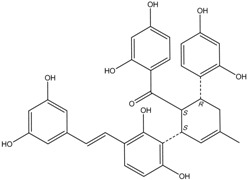	15.5 ± 0.8	16.2 ± 0.7
J14715 Albafuran C	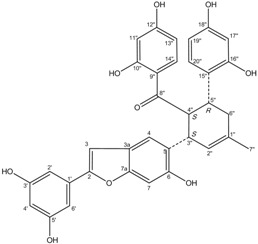	11.9 ± 0.7	10.7 ± 0.6
J14716 Mulberrofuran J	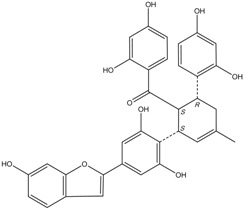	18.4 ± 1.4	25.4 ± 2.1

*: IC_50_ was performed with purified ADAMTS1 expressed in the *E. coli*; ^#^: IC_50_ was performed with purified ADAMTS1 expressed in the mammalian cells.

Obviously, further investigation is needed to study the compounds’ inhibitory activities towards structurally distinct substrates and other ADAMTS family members, to elucidate the structure/inhibition relationship of these compounds, and reveal the inhibiting mechanism in the molecular level.

**Figure 6:**
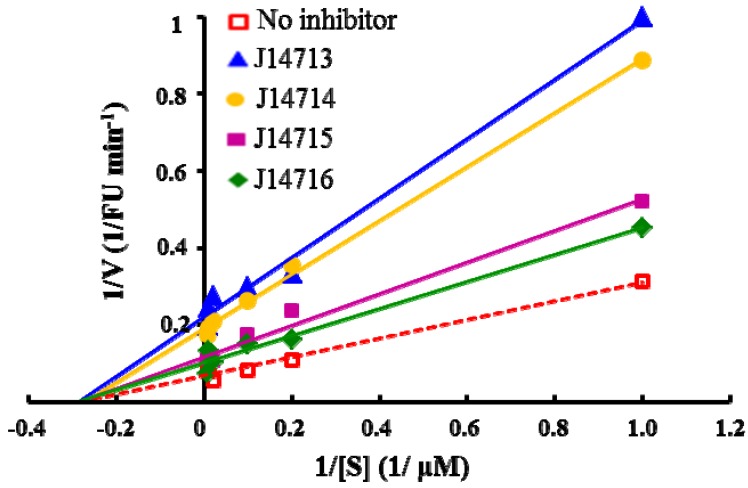
Lineweaver-Burk plots of enzyme kinetics of ADAMTS1 inhibitors. Each inhibitor was tested in triplicate in two independent assays at the concentration of 10 µg/mL. All four compounds only show decrease in *V*max without a change in *K*m, indicating noncompetitive inhibition.

The experiments to explore the mode of inhibition by these four compounds were performed by measuring the fluorescence increase at various substrate concentrations (0, 10, 25, 50, 100 and 150 µM) in the absence and presence of inhibitors. The mode of inhibition was determined separately by measuring kinetic parameters and by plotting the data in the Lineweaver-Burk plots. Our preliminary kinetic studies indicated that all four inhibitors exhibited noncompetitive inhibition, with a decrease in *V*max, but no effect on *K*m, shown in Figure 6. The noncompetitive inhibition of ADAMTS1 suggests that these four hits do not bind to the active site of ADAMTS1 but may act as an allosteric inhibitor at a distinct site in the ADAMTS1 molecule. Further structural studies may help to elucidate the precise interaction site where these inhibitors bind to the ADAMTS1 protein and give insights into the mode of action of those inhibitors.

## 3. Experimental

### 3.1. Materials

*E. coli* BL21 (DE3), *E. coli* DH5a, pET32a(+) vector and his-bind purification kit were purchased from Novagen. Heparin sepharose CL-6B column was purchased from Amersham Biosciences. The peptide, Abz-YPLPRNITEGEARGNVILTAK(Dnp)P-OH, was synthesized by GL Biochem Ltd. (Shanghai, China). Recombinant human ADAMTS1 was purchased from R&D Systems. Other reagents and solvents used were of analytical grade. The fluorescence was measured by SpectraMax M5 Multi-Mode Microplate Reader. All samples and buffers were added into 384-well plates using Biotec model EDR-96S/384S Multi Dispenser.

### 3.2. Cloning, Expression and Purification of the Recombinant ADAMTS1

The 1452 nt cDNA fragment (1206-2657) of human ADAMTS1 (GenBank accession no. 50845383) was cloned using polymerase chain reaction (PCR) method. The forward primer (5’-GCGGGATCC*GACGACGATGACAAA*AAGCGATTTGTGTCCAGTCA-3’) contained a site for restriction enzyme BamHI (underlined) and a nucleotide acid sequence (italicized) encoding the recognition site of enterokinase (Asp-Asp-Asp-Asp-Lys-). The reverse primer (5’-GCCCTCGAG*AATGATGATGATGATGATGAT*TGCACTAGTAACTGATCCTGATAT-3’) carried a site for restriction enzyme XhoI (underlined), a termination codon and a nucleotide acid sequence encoding His·tag (italicized). The PCR products were ligated to expression vector pET32a(+) (Novagen, USA) and transformed into competent *E. coli* BL21. The recombinant pET32a-ADAMTS1 was confirmed by sequencing. The positive colonies were grown in fresh LB (200 mL) with 100 g ampicillin/L. 1 mM IPTG was added at the OD_600_ of approximately 0.6 and the induction was performed at 20 °C for 12 h. The induced cells were harvested and lysed by ultrasonication in the Ni-NTA binding buffer. After centrifugation at 10,000 g for 10 min the supernatant was applied to a Ni-NTA bind resin column. The peak fractions containing the target fusion proteins were collected. Then 1 unit enterokinase was added into 1,000 μg of purified fusion protein ADAMTS1. After incubation at 25 °C for 15 h, the proteolytic reaction was terminated by adding PMSF up to 1 mM. The recombinant ADAMTS1 was further purified using heparin-sepharose CL-6B column and analyzed using SDS-PAGE and Western blotting with ADAMTS1 antibody. The intensity of protein bands was quantified using a densitometer (Bio-Rad).

### 3.3. ADAMTS1 Activity Assay

To establish a rapid and sensitive activity assay for the high-throughput screening of ADAMTS1 inhibitors, we designed a FRET peptide, Abz-YPLPRNITEGEARGNVILTAK(Dnp)P-OH, as the substrate, which contained a fluorescent group (*o*-aminobenzoic acid, Abz ) and a quencher group (2,4-dinitrophenyl, Dnp) as a donor-acceptor pair at the N and C terminus of the peptide, respectively. The substrate peptides were incubated in 100 µL solution containing 20 mM Tris-HCl, pH 7.5, 100 mM NaCl, 10 mM CaCl_2_ at 37 °C for 3 h in the presence of recombinant ADAMTS1. The substrate peptides without the recombinant ADAMTS1 were incubated in the same solution and used as control. The fluorescence emitted by the cleaved substrate was measured by SpectraMax M5 Microplate Reader (320 nm excitation, 420 nm emission).

### 3.4. Effects of pH, Temperature and DMSO Solvent on the ADAMTS1 Activity

The optimal pH for the activity of ADAMTS1 was determined at 37 °C with 30 min incubation. Intervals of 1 pH unit were used with the pH range of 5 to 11. The buffer containing 20 mM Tris-HCl, 100 mM NaCl, 10 mM CaCl_2_ was used and the buffer pH was adjusted to 5–11 by adding either HCl or NaOH solution. The optimal temperature for the enzymatic activity was determined with temperature range from 20 to 50 °C at the optimal pH. The results were showed as percentages of the activity obtained at either the optimal pH or temperature. The effect of DMSO on the enzyme activity was investigated by incubating the enzyme for 30 min at 37 °C in buffers containing 1% (v/v) DMSO at the optimal pH and temperature.

### 3.5. Determination of the Kinetic Parameters Using the FRET Peptide

The kinetic parameters *K*m and *V*max of purified ADAMTS1 were determined by monitoring the fluorescence change. They were measured under standard assay conditions at 0, 10, 25, 50, 100 and 150 µM peptide substrate concentrations in the absence and presence of 10 nM ADAMTS1. Assay mixtures without enzymes were served as controls. The initial velocity for the cleavage of substrate was determined from the slope of the initial part of the emission versus time curve at each concentration. The kinetic parameters *K*m and *V*max were determined from linear regression analysis of plots of the inverse of the reaction velocity versus the inverse of the substrate concentration (Lineweaver-Burk plot). We compared the kinetic parameters of mammalian cell-expressed ADAMTS1 under the same assay conditions. The inhibition mode of ADAMTS1 by the hits was determined kinetically using 10 nM commercial ADAMTS1 at various substrate concentrations (0–150 µM) in the absence and presence of 10 µg/mL inhibitors. 

### 3.6. High-Throughput Drug Screening

To validate the HTS assay, the coefficient of variation (CV), the signal to background value (S/B), and the Z’ factor were calculated from a single black 384-well microplate including the negative controls (absence of ADAMTS1, n = 192) and the positive controls (presence of ADAMTS1, n = 192). According to Zhang’s method [24], the assay performance was evaluated by the following equation (1):

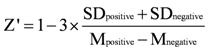
(1)
where M is the mean fluorescence value for controls, and SD is the standard deviation. The minimal acceptable value for a high-throughput screening assay is usually considered to be 0.5. The theoretical maximum is 1.

High-throughput screening was performed against the diverse library (40,960 total compounds) from the National Compound Resource Center (Beijing, China). Compounds were preplated in 1% DMSO at a final concentration of 10 µg/mL. 5 µL of each compound was added into 45 µL optimized reaction system containing 20 mM Tris-HCl, 100 mM NaCl, 10 mM CaCl_2_, 3.38 µM peptide substrate and 10 nM ADAMTS1 enzyme in a black 384-well microplate. There were 376 test compounds, four negative controls (absence of ADAMTS1) and four positive controls (presence of ADAMTS1) on each plate. All the samples and buffers were dispensed into each of 384 wells using Biotec model EDR-96S/384S Multi Dispenser. The incubation time of ADAMTS1 catalyzed reaction was 30 min at 37 °C and the fluorescence was measured by SpectraMax M5 Microplate Reader as described above. The inhibition percentage of ADAMTS1 was calculated by equation (2) [25]:

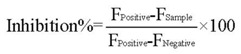
(2)
where F is the measured fluorescence. Compounds showing more than 60% inhibition in the primary screening were identified and further performed secondary screening under the same condition to limit errors possibly occurred. A dose-response experiment was performed using the purified ADAMTS1 expressed in *E. coli* and mammalian cell line to confirm the inhibitory capacity of these HTS hits against ADAMTS1. The concentrations resulting in 50% inhibition were calculated using the sigmoidal dose-response equation in the GraphPad Prism software.

## 4. Conclusions

We have developed a FRET assay for ADAMTS1 activity detection, which is sensitive, reliable, and convenient. This assay is not only suitable for the quantitative assay of ADAMTS1 activity, but also useful for high-throughput screening of its inhibitors. To the best of our knowledge, it is the first reported HTS system targeting ADAMTS1, which is expected to expedite the discovery of novel ADAMTS1 inhibitors in the future. The novel ADAMTS1 inhibitors identified from the screening will be further tested for selectivity against ADAMTS-4/5 and MMP-12/13, other metalloproteases implicated in the OA. These compounds may be further modified and tailored by chemical methods and served as therapeutic candidates in ADAMTS1-related drug discovery research.
